# Peer Review of “COVID-19 and Cybersecurity: Finally, an Opportunity to Disrupt?”

**DOI:** 10.2196/29417

**Published:** 2021-05-06

**Authors:** David W Chadwick

**Affiliations:** 1 University of Kent Kent United Kingdom

**Keywords:** COVID-19, cybersecurity, challenges and disruption, data protection, privacy, health data

*This is a peer-review report submitted for the paper "COVID-19 and Cybersecurity: Finally, an Opportunity to Disrupt?"*

## Round 1 Review:

### General Comments

This paper [1] provides an overview of cybersecurity and privacy risks arising from COVID-19. Overall, the paper is quite generic, and I did not think that it identified any new issues or presented any new results.

### Specific Comments

#### Major Comments

1. When discussing data sharing privacy versus public health for the contact tracing apps, why didn't the author compare what dictatorial regimes such as China are doing at one extreme (eg, recording every railway carriage that someone travels in) with the more privacy-protecting method championed by Google and Apple?
2. When discussing fraud and theft, why didn’t the author mention the fact that Google still accepts “paid” advertisements from fraudulent financial services and publishes these at the top of search results, even though Google has known about this problem for years?

#### Minor Comments

There are a few grammatical errors, as follows:

1. “time is of essence” should be “time is of the essence”
2. “elder people” should be “elderly people” (twice)
